# Emergence of KPC-8-producing *K. pneumoniae* infection without prior exposure to ceftazidime/avibactam: the threat of *de novo* infections by ceftazidime/avibactam-resistant KPC variants

**DOI:** 10.1128/aac.01494-24

**Published:** 2025-04-22

**Authors:** María Alejandra Mateo-Vargas, Salud Rodríguez-Pallares, Jorge Arca-Suárez, Lorena López-Cerero, Manuel Rodríguez-Iglesias, Fátima Galán-Sánchez

**Affiliations:** 1 Departamento de Biomedicina, Biotecnología y Salud Pública, Universidad de Cádiz, Cadiz, Spain; 2 Servicio de Microbiología and Instituto de Investigación Biomédica A Coruña (INIBIC), Complexo Hospitalario Universitario A Coruña, A Coruña (Spain), A Coruña, Spain; 3 CIBER de Enfermedades Infecciosas, Instituto de Salud Carlos III, Madrid, Spain; 4 Unidad Clínica de Enfermedades Infecciosas y Microbiología, Hospital Universitario Virgen Macarena, Instituto de Biomedicina de Sevilla IBIS, Seville, Spain; 5 Departamento de Microbiología, Universidad de Sevilla, Seville, Spain; 6 Servicio de Microbiología and Instituto de Innovación e Investigación Biomédica de Cádiz (INIBICA), Hospital Universitario Puerta del Mar, Cadiz, Spain; Universita degli studi di roma La Sapienza, Rome, Italy

**Keywords:** KPC-8, *Klebsiella pneumoniae*, ceftazidime/avibactam, KPC-31, meropenem/vaborbactam, evolution, imipenem/relebactam, cefiderocol, β-lactamase, antimicrobial resistance

## Abstract

*De novo* infections caused by ceftazidime/avibactam-resistant KPC variants are rarely reported. We characterize the evolution of a KPC-8-producing *Klebsiella pneumoniae* strain involved in a primary infection without previous ceftazidime/avibactam treatment. During a 15-month follow-up, changes in carbapenem susceptibility due to porin alterations were observed, remaining susceptible to meropenem/vaborbactam, imipenem/relebactam, and cefiderocol. High- and low-permeability recombinant *Escherichia coli* isolates analysis revealed that, unlike the widespread ceftazidime/avibactam-resistant variant KPC-31, KPC-8 confers ceftazidime/avibactam resistance without decreasing carbapenemase activity.

## INTRODUCTION

Ceftazidime/avibactam has proven activity against multidrug-resistant bacteria. We describe the emergence of a primary infection caused by carbapenem- and ceftazidime/avibactam-resistant KPC-8-producing *Klebsiella pneumoniae* (KPC-8-*Kp*) in a single patient and characterize its evolution, genomic trade-offs, and phenotypic features via whole genome sequencing (WGS) and cloning experiments. We also provide a comparison between KPC-8, KPC-3, and KPC-31 and their interplay with decreased outer membrane permeability using recombinant *Escherichia coli* isolates.

**Fig 1:**
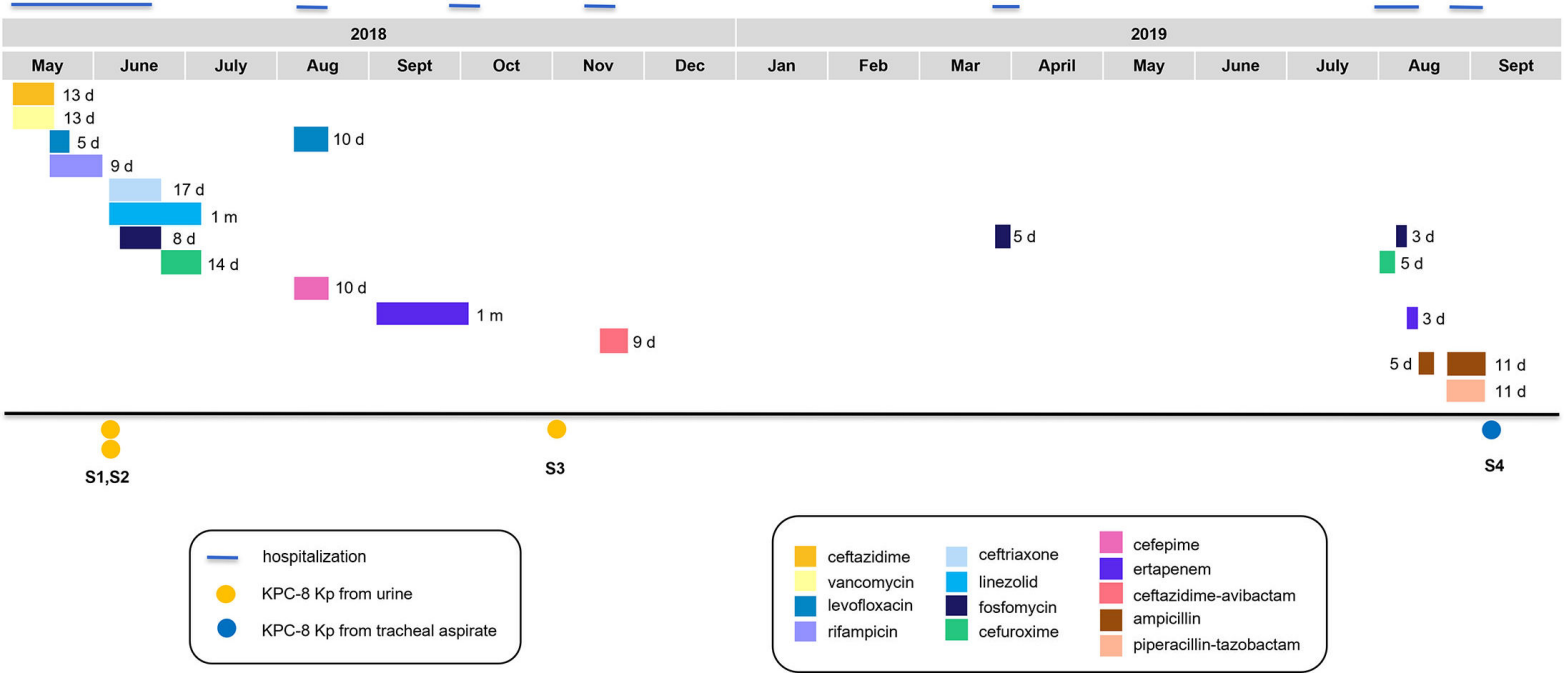
Timeline of consecutive KPC-8-Kp clinical isolates obtained from the patient during the period 2018–2019. Colored blocks—displayed following the same order as they appear in the key—represent the antibiotic uptake and the length of the treatment (days). Blue lines on top represent the length of hospitalization.

Four KPC-*Kp* isolates (S1–S4) were recovered from a 35-year-old male from June 2018 to September 2019. Before S1 and S2 were recovered from urine, ceftazidime/avibactam was not prescribed (Fig. 1). MIC values for both isolates were identical (Table 1). S3 and S4 were isolated in October 2018 and September 2019 from urine and tracheal aspirates, respectively. Nine months before S4 was recovered, the patient received ceftazidime/avibactam treatment (Fig. 1). Both isolates showed the same antimicrobial susceptibility pattern as S1 and S2, except for imipenem and meropenem (*I* = susceptible with increased exposure, according to EUCAST definitions).

**TABLE 1:** Antimicrobial susceptibility testing of S1 and S4 clinical isolates, *E. coli* TG1 and *E. coli* HB4 (*ompF* and *ompC*-deficient) recombinant isolates expressing KPC-3, KPC-8, and KPC-31 β-lactamases^*d*^

Strain	MIC (mg/L) ^*a*^											
	TZP (*R* > 8)	ATM (*R* > 4)	CTX (*R* > 2)	CAZ (*R* > 4)	CZA (*R* > 8)	FEP (*R* > 4)	FDC (*R* > 2)	ETP (*R* > 0.5)	IPM (*R* > 4)	I/R (*R* > 2)	MEM (*R* > 8)	MVB (*R* > 8)
S1 **^*b*^**	>64	>64	>64	>64	16	>64	1	>64	32	0.125	64	1
S4 ^*c*^	>64	>64	64	>64	16	64	1	16	4	≤0.06	4	≤0.06
*E. coli* TG1	1	≤0.06	≤0.06	0.25	≤0.06	≤0.06	≤0.06	≤0.06	≤0.06	≤0.06	≤0.06	≤0.06
*E. coli* TG1 + pUCP24- KPC-3	>64	64	4	32	0.25	4	≤0.06	4	2	≤0.06	2	≤0.06
*E. coli* TG1 + pUCP24- KPC-8	>64	>64	64	>64	16	32	0.5	1	2	≤0.06	0.5	≤0.06
*E. coli* TG1 + pUCP24- KPC-31	4	0.5	0.25	64	16	1	0.25	≤0.06	0.125	≤0.06	≤0.06	≤0.06
*E. coli* HB4	16	0.125	0.25	1	0.5	0.5	≤0.06	≤0.06	≤0.06	≤0.06	≤0.06	≤0.06
*E. coli* HB4 + pUCP24- KPC-3	>64	>64	32	>64	4	>64	0.25	16	32	0.125	64	0.25
*E. coli* HB4 + pUCP24- KPC-8	>64	>64	>64	>64	64	>64	1	16	16	0.125	32	0.25
*E. coli* HB4 + pUCP24- KPC-31	16	4	8	>64	64	64	1	0.25	0.25	0.125	0.5	0.25


^*a*^ 2024 EUCAST BREAKPOINTS INDICATED.

^*b*^ S1 and S2 showed the same antimicrobial susceptibility pattern.

^*c*^ S3 and S4 showed the same antimicrobial susceptibility pattern.

^*d*^ TZP, piperacillin/tazobactam; ATM, aztreonam; CTX, cefotaxime; CAZ, ceftazidime; CZA, ceftazidime/avibactam; FEP, cefepime; FDC, cefiderocol; ETP, ertapenem; IPM, imipenem; I/R, imipenem/relebactam; MEM, meropenem; MVB, meropenem/vaborbactam.

**Fig 2:**
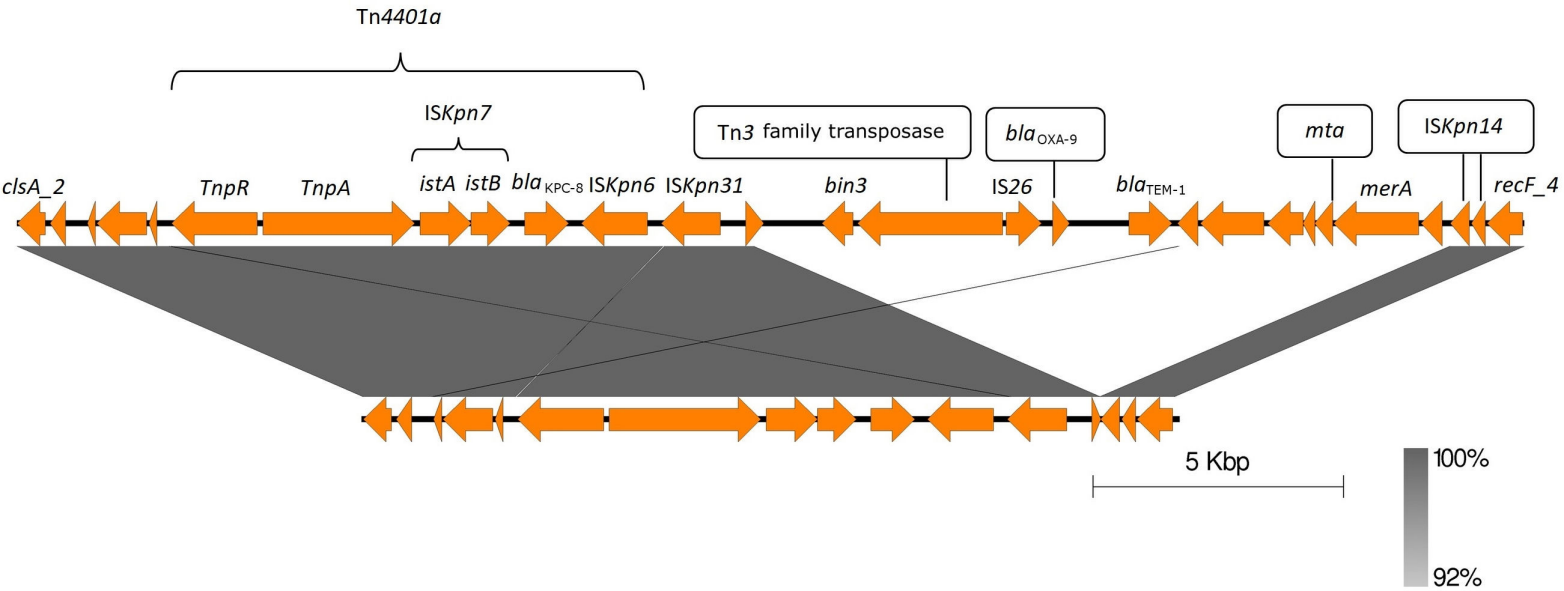
*bla*_KPC-8_ genetic environment comparison of S1 (top) and S4 (bottom). A large deletion (13,483 bp) in S4—leading to the loss of several genes including *bla*_TEM-1_—is observed, as well as some gene reorganization. Obtained using Easyfig (2).

To investigate clonal relatedness and underlying resistance mechanisms, all isolates were sequenced (MiSeq, Illumina Inc., San Diego, USA). S1 and S4 were additionally sequenced via MinION Mk1C (Oxford Nanopore Technologies) to obtain their hybrid assemblies. All the isolates belonged to the sequence type ST512, cgMLST 18404, serotype KL107, O1/O2v2, and capsular type wzi154. Antimicrobial determinants and chromosomal point mutations are described in Table 2. All the isolates harbored *bla*_KPC-8_. This minority variant of KPC-3 involves the V240G amino acid change (1). Both the *ompK35* and *ompK37* porin genes contained premature stop codons. In S1 and S2, p.134insGD was detected in *ompK36*. None of the isolates carried hypermucoviscosity-related genes (*magA*, *rmpADC/A2)*, toxins, or yersiniabactin- or allantoin-producing genes. Virulence genes are detailed in Table S1. All the isolates carried the ColRNAI, IncFIB(K), IncFII(K), and IncFIB(pQil) replicon sequences. S1 and S4 contained a ColRNAI (13,636 bp) and an IncFIB(K)/IncFII(K)/IncFIB(pQil) (236,610 bp for S1 and 223,127 bp for S4) plasmid (Table 2). The IncF plasmid of S1 carried the resistance genes *bla*_KPC-8_, *bla*_TEM-1_, *dfrA12*, *catA1*, *qacE*, *mph(A)*, *sul1*, *aph(3’)-la*, and *aadA2. bla*_TEM-1_ was transported by a composite transposon, and *bla*_KPC-8_ was transported by a Tn4401a transposon. The *bla*_KPC-8_ genetic environment was the same in all isolates. The IncF plasmid presented a large deletion (13,483 bp) in S4, leading to the loss of several genes including *bla*_TEM-1_ (Fig. 2). Phylogenetic analysis revealed a distance of <2.2 SNPs/month between isolates from consecutive samples (Table S2).

**TABLE 2:** Genotypic traits of KPC-8-producing *K. pneumoniae* isolates included in this study^*d*^

Isolate	ST	Capsular			Antimicrobial determinants		Porins			Rep seq
		Type (wzi)	LPS ^*a*^ (*O*)	PS ^*b*^ (*K*)	β-lactamases	Others	OmpK35	OmpK36	OmpK37	
**S1**	512	154	O1/O2v2	KL107	*bla*_KPC-8_; *bla*_SHV-11_; *bla*_TEM-1A_; *bla*_OXA-9*_	*fos*A*6; aac(6')-lb; aph(3')-la; aadA2; mph(A); oqxA; oqxB; catA1; qacE; sul1*	E89*	134insGD	K251^*c*^	ColRNAI; IncFIB(K); IncFII(K); IncFIB(pQil)
**S2**					*bla*_KPC-8_; *bla*_SHV-11_; *bla*_TEM-1A_; *bla*_OXA-9*_			134insGD		
**S3**					*bla*_KPC-8_; *bla*_SHV-11_; *bla*_TEM-1A_; *bla*_OXA-9*_			wild type		
**S4**					*bla*_KPC-8_; *bla*_SHV-11_			wild type		


^*a*^ LPS: lipopolysaccharide.

^*b*^ PS: polysaccharide.

^*c*^ Premature stop codon.

^*d*^ ST: sequence type.

To analyze the impact of the V240G amino acid substitution on the β-lactam resistance profile of the KPC enzyme and its interplay with low outer membrane permeability, KPC-8 was cloned in parallel with the wild-type KPC-3 and the widespread ceftazidime/avibactam-resistant variant KPC-31 under low- and high-permeability backgrounds, following our previously described methodology (3). Comparative phenotypic data for these variants are shown in Table 1.

**Fig 3:**
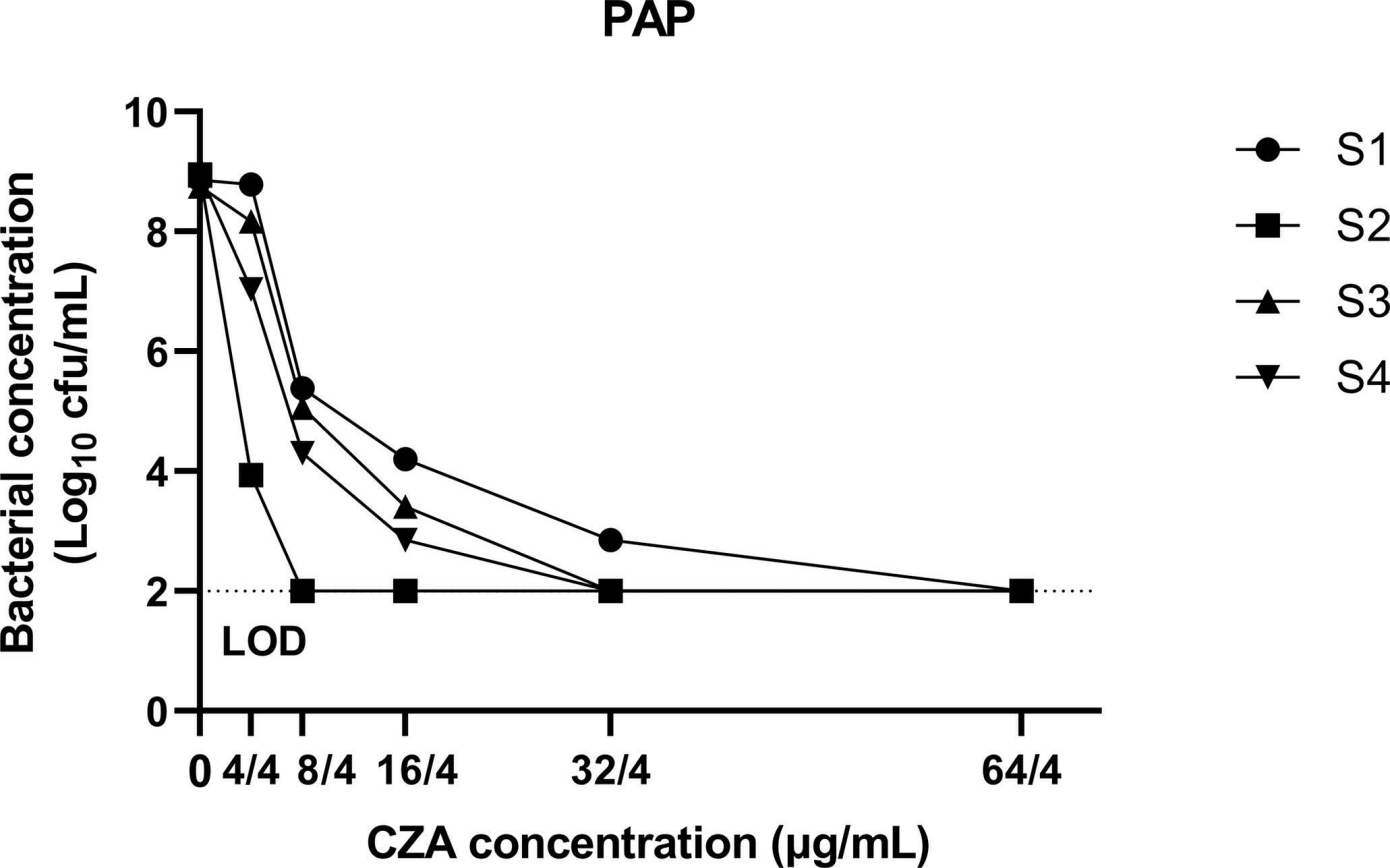
Population analysis profile for all isolates at the selected five concentrations of ceftazidime/avibactam (CZA). Bacterial concentration is represented as log_10_ CFU/mL.

Population analysis profiling showed a decrease in all bacterial populations since the first concentration of ceftazidime/avibactam tested (4/4 mg/L), except for S1 (Fig. 3; Table S3). Bacterial reduction of between 4- and 6-log_10_ CFU/mL was noticed for S1, S3, and S4 at 16/4 mg/L of ceftazidime/avibactam. Additionally, growth at 32/4 mg/L for S1 was observed. These results suggest the presence of more than one bacterial population.

**Fig 4:**
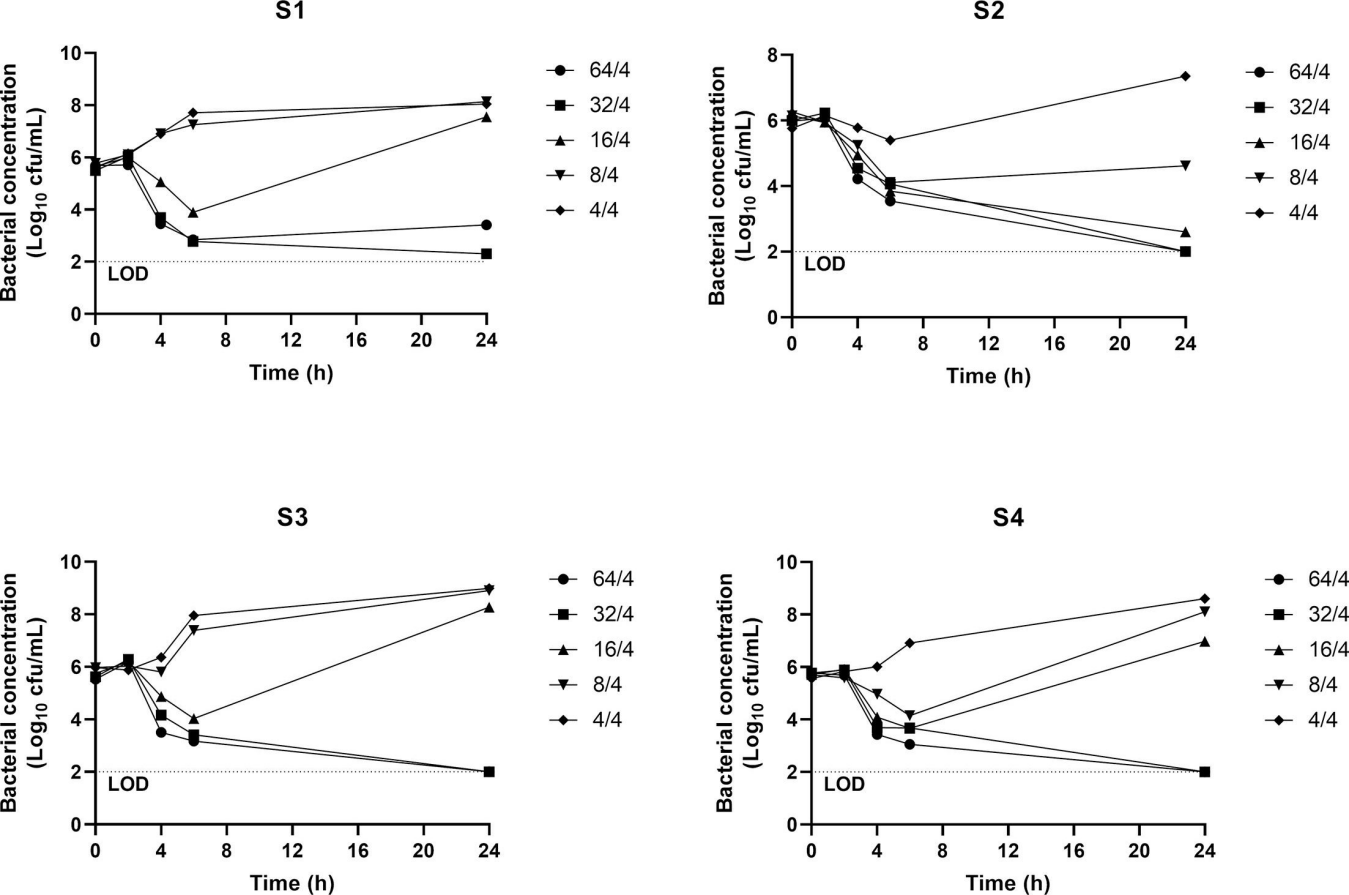
Time-kill assay results for the four isolates at the selected five concentrations of ceftazidime/avibactam (64/4, 32/4, 16/4, 8/4, and 4/4 mg/L). Bacterial concentration (log_10_ CFU/mL) is represented at 0, 2, 4, 6, and 24 h.

A time-kill assay was performed (4). S1, S2, and S4 outgrew the MIC previously determined by microdilution (16/4 mg/L), growing at 16/4, 32/4 (S1 and S2), and 64/4 mg/L (S1 and S3; Fig. 4, Table S4). The regrowth after 24 h of incubation at 16/4 mg/L of ceftazidime/avibactam suggests the coexistence of more than one population. For S2, the presence of two subpopulations can be concluded at 4/4 mg/L. The most resistant subpopulations (growing at ≥16/4 mg/L) were sequenced (Illumina Inc, San Diego, USA) and compared to the original strains (MIC = 16/4 mg/L). No SNPs were found coding for β-lactamases, porins, efflux pumps, or PBP-related genes (Table S5).

To date, few studies have investigated KPC-8. An outbreak associated with this carbapenemase was reported in Puerto Rico in 2008 (5). However, KPC-8 was not detected in a recently published large Spanish multicenter study (6). Thus, this is the first report of this variant in Spain. In addition to characterizing this variant and its intrapatient evolution, this study presents a case of KPC-8-*Kp* infection in a patient with no previous ceftazidime/avibactam treatment.

In our study, *bla*_KPC-8_ is carried by a Tn4401a transposon nearly identical to NZ_JAMQEI010000039.1, except for the KPC variant (7). To date, we have not found studies describing the *bla*_KPC-8_ genetic environment. However, Tn4401a transposons are well known (8), and they often carry *bla*_KPC-2_ (9) and *bla*_KPC-3_ genes (10). The KPC-8 amino acid substitution (V240G) is located close to the active site, where it enlarges and strongly contributes to ceftazidime hydrolysis (1), making this variant resistant to ceftazidime/avibactam (11, 12). Like KPC-31, KPC-8 exhibited increased resistance to ceftazidime/avibactam, including subpopulations able to grow at concentrations as high as 64/4 mg/L (Fig. 4). However, KPC-8 expression increased the MICs of imipenem and meropenem in a similar way as KPC-3 did, resulting in higher values than those observed for KPC-31. This finding provides evidence that this substitution does not collaterally result in carbapenem susceptibility. These values significantly increased in porin-deficient recombinant isolates, revealing that KPC-8 may confer higher carbapenem MIC values under low-permeability conditions, a feature shared with KPC-3 and KPC-31. This would explain the lower MICs for carbapenems among the clinical isolates displaying wild-type porins (S3 and S4) compared to those carrying the p.134insGD in OmpK36 (S1 and S2) (13). Major *ompK36* mutations in KPC-*Kp* isolates reportedly result in extremely high carbapenem MICs (14) and emerge under meropenem selection *in vitro* (14). Nevertheless, the patient did not receive meropenem treatment prior to (or after) the first sampling, and surprisingly, according to the chronology of the isolations, the mutants preceded the wild-type isolates. Porin-mutated bacteria have been described to have less fitness than their isogenic parental strains expressing wild-type OmpK36, which could explain the presence of the wild-type population (S3 and S4) by the end of the follow-up (15).

In terms of new antimicrobials that remain active, meropenem/vaborbactam shows low MIC values against ceftazidime/avibactam-resistant KPC-producing isolates, in agreement with those reported by Wilson et al*.* (11). Vaborbactam passage across the outer membrane seems to be more affected by mutations in *ompK36*, which has a smaller channel than OmpK35 does. Consistent with Wilson et al*.* findings, meropenem/vaborbactam MICs were slightly higher in KPC-*Kp* isolates with a mutant *ompK36* than in those with wild-type *ompK36*. In our study, KPC-8-*Kp* was susceptible to imipenem/relebactam, which has been proven to be active against ceftazidime/avibactam-resistant KPC-*Kp* (16). Cefiderocol has potent *in vitro* activity against KPC-producing Enterobacterales, mostly KPC-2 and KPC-3 (17), although we have not found previous studies testing cefiderocol against KPC-8-*Kp*. Cross-resistance due to structural similarities with ceftazidime has been reported (18). In our study, all the isolates were susceptible to this antimicrobial agent, although KPC-8- and KPC-31-producing transformants showed higher MICs than those expressing KPC-3.

In conclusion, this study revealed that the emergence of ceftazidime/avibactam-resistant KPC variants can lead to infections without previous ceftazidime/avibactam selection. Thus, we could face a problematic therapeutic challenge. As for KPC-8, only cefiderocol, meropenem/vaborbactam, and imipenem/relebactam remain therapeutic options. This highlights the importance of monitoring and controlling the dissemination of new variants.

## Data Availability

This Whole Genome Shotgun project has been deposited at DDBJ/ENA/GenBank under the Bioproject accession number PRJNA1026946.

## SUPPLEMENTAL MATERIAL

Supplemental material aac.01494-24-s0001.docx Tables S1 to S4; Supplemental text.

Table S5 aac.01494-24-s0002.xlsx SNPs found among the isolates that grew above the MIC vs the original sequenced isolates (MIC = 16 mg/L).

ASM does not own the copyrights to Supplemental Material that may be linked to, or accessed through, an article. The authors have granted ASM a non-exclusive, world-wide license to publish the Supplemental Material files. Please contact the corresponding author directly for reuse.
