# Supplementary material for: Emergence of KPC-8-producing K. pneumoniae infection without prior exposure to ceftazidime/avibactam: the threat of de novo infections by ceftazidime/avibactam-resistant KPC variants
Source: Antimicrob Agents Chemother. 2025 Apr 22;69(6):e01494-24. doi: 10.1128/aac.01494-24 (PMC12135529; doi:10.1128/aac.01494-24)
Supplement: Supplemental material — Tables S1 to S4; Supplemental text. [file aac.01494-24-s0001.docx]

**SUPPLEMENTARY INFORMATION**

**Case description**

Four KPC-*Kp* isolates were recovered from a 35-year-old male from June 2018 to September 2019. All of them were immediately preserved after their sampling at -80ºC in the strain collection of the Hospital Universitario Puerta del Mar (HUPM) in Cadiz, Spain. The patient suffered from Behçet’s disease, iatrogenic Cushing’s syndrome and was treated with multiple immunosuppressive therapies. One month after the diagnosis of an osteosynthesis implant infection in May 2018, the patient was treated with a combination of four antibiotics, and S1 and S2 were recovered from urine (**Figure 1**). No treatment with ceftazidime/avibactam was prescribed prior these isolates sampling. S3 and S4 were isolated in October 2018 and September 2019 from urine and tracheal aspirates, respectively. The patient required multiple admissions and antibiotic treatments during the study period and passed away in September 2019 due to multi-organ failure (**Figure 1**).

**Genetic analysis**

Genomic DNA was extracted with EZ1 Advanced XL (Qiagen). Paired-end libraries were obtained using the Nextera XT DNA kit (Illumina Inc, San Diego, USA) and sequenced on a MiSeq Illumina instrument (Illumina Inc., San Diego, USA) with 600 cycle MiSeq Reagent Kit v3, following the protocol Illumina DNA Prep Reference Guide (Document # 1000000025416 v09, Illumina Inc). The depth coverage was 45x for S1, 42x for S2, 38x for S3 and 62x for S4. The obtained reads quality was assessed by FastQC (1) and they were trimmed using Trimmomatic-v.0.36 (2). *De* *novo* assembly (SPAdes-v.3.13.0, (3)) and annotation (RASTtk,(4)) of the genome were performed with manual correction. Outer membrane porins sequences were aligned to identify differences between the isolates (CLC Genomics Workbench-v12.0 (QIAGEN, Les Ulis, France)). Typing with PathogenWatch-v12.0.4 (https://pathogen.watch/), resistome (ResFinder-v4.1 <http://genepi.food.dtu.dk/resfinder>, CARD-v3.2.4 (5)), virulome (VFDB (6)), plasmidome (PlasmidFinder-v2.1 (<https://cge.food.dtu.dk/services/PlasmidFinder/>), pMLST-v2.0 (<https://cge.food.dtu.dk/services/pMLST/>)) and SNPs (CSI Phylogeny-v1.4 <https://cge.food.dtu.dk/services/CSIPhylogeny/>, being S1 used as a reference genome and setting the default parameters) analyses were performed. For the plasmidome study, S1 and S4 were sequenced via MinION Mk1C with a R9.4.1 flow cell, following the Rapid Barcoding kit protocol (Oxford Nanopore Technologies). The obtained contigs were trimmed via Porechop (Galaxy Version 0.2.4+galaxy0; (7)) with default parameters. Thus, hybrid assemblies were obtained via Unicycler (Galaxy Version 0.5.0+galaxy1; (8)). Prokka (toolshed.g2.bx.psu.edu/repos/crs4/prokka/prokka/1.14.6+galaxy1) was used to annotate the genomes. For S1 and S4, contig 2 was a IncFIB(K)/IncFII(K)/IncFIB(pQil) multireplicon plasmid, being contig 1 and contig 3 the bacterial chromosome and a ColRNAI plasmid, respectively. The *bla*_KPC-8_ genetic environment was compared by Easyfig-v.2.2.5 (9), with S2 and S3 Illumina contigs aligned and compared via CLC Genomics Workbench-v.12.0 (QIAGEN). Short-reads were aligned against the *bla*_KPC-8_ sequence for each isolate (CLC Genomics Workbench-v12.0 (QIAGEN)), discarding the presence of KPC-3-producing subpopulations. Outer membrane porin sequences were aligned to identify differences between the isolates (CLC Genomics Workbench-v12.0 (QIAGEN)).

**Construction of recombinant *E. coli* isolates expressing *bla*_KPC_ under high and low permeability conditions**

The *bla*_KPC-3_, *bla*_KPC-8_ and *bla*_KPC-31_ genes were amplified in parallel by PCR, with primers 5’ - CGAGCTCGCCATGCCCATATCCTGACCCTG - 3’ and 5’- CGGGATCCGCCGCGCAGACTCCTAGCCTAAA - 3’. The PCR products were ligated to plasmid pUCP24 using SacI and BamHI restriction sites to obtain plasmid pUCP24-KPC, which was electroporated in parallel into the *E. coli* TG1 and into the OmpC- and OmpF-deficient *E. coli* HB4 strains, according to previously described protocols (10). Transformants were selected on LB agar plates containing 10 mg/L-gentamicin. Recombinant isolates were characterized by MIC determination following the methodology described below.

**Antimicrobial Susceptibility Testing**

Minimum inhibitory concentrations (MICs) of piperacillin/tazobactam, aztreonam, cefotaxime, ceftazidime, ceftazidime/avibactam, cefepime, cefiderocol, ertapenem, imipenem, imipenem/relebactam, meropenem and meropenem/vaborbactam were determined for clinical isolates and transformants in triplicate experiments by reference broth microdilution assays. The MICs were determined using cation-adjusted Müeller-Hinton (MH) broth in all cases, with the exception of cefiderocol, which was assessed using iron-depleted cation-adjusted MH broth prepared according to CLSI M100 guidelines (11). Tazobactam, avibactam and relebactam were tested at a fixed concentration of 4 mg/L, whereas vaborbactam was tested at 8 mg/L. EUCAST-v14.0 clinical breakpoints and guidelines (<https://www.eucast.org/clinical_breakpoints>) were used for reference purposes. The reference strains *E. coli* ATCC 25922, *E. coli* NCTC 13353 and *K. pneumoniae* ATCC BAA-2814 were used as a control.

**SNPs analysis**

The SNPs comparison among the clinical isolates is represented in **Supplementary Table 2**, with S1 as a reference genome. The percentage of the reference genome covered by all isolates is 99.72%. In a period of almost 5 months the number of SNPs between the first strains (S1, S2) and the one isolated in a consecutive sample (S3) is 9 and 11 respectively. This is 1.8 and 2.2 SNPs per month. The difference between S3 and S4 is 19 SNPs in 10 months, being 1.9 SNPs per month. All the isolates from consecutive sampling showed a similar mutation rate per month.

**Population Analysis Profiling (PAP)**

Population Analysis Profiling (PAP) was performed to determine the coexistence of different subpopulations within the four samples. The preinoculum of each isolate – comprising single overnight colonies cultivated in Mueller-Hinton II broth and incubated at 37ºC with shaking – was diluted 1/10^5^ and was also incubated overnight under the same conditions. Serial dilutions of the starting inoculums were cultivated in MHA plates at five concentrations of ceftazidime (4, 8, 16, 32 and 64 mg/L) with a fixed concentration of avibactam (4 mg/L). After 24h of incubation at 37ºC, the number of colonies was counted. The frequency of bacteria resistant to each antimicrobial concentration appearance was calculated by dividing the average number of colonies counted at a given ceftazidime/avibactam concentration by the average number of colonies that grew in the absence of this antimicrobial (**Supplementary Table 3**).

**Time-kill assay**

A time-kill assay was performed for the four strains at five concentrations of ceftazidime (4, 8, 16, 32 and 64 mg/L) with a fixed concentration of avibactam (4 mg/L) as described by Docobo *et al.* (12). The starting inoculums were set at 5x10^5^ CFU/mL. The number of viable CFU was determined at 0, 2, 4, 6 and 24h by serial dilution. Bacterial cultures were cultivated on MHA plates and after 24h of incubation at 37ºC, the number of colonies was counted. The most resistant subpopulations were sequenced through the short-read approach previously described (Illumina Inc, San Diego, USA). Thus, up to 3 colonies growing at a concentration of ≥16/4 mg/mL (≥MIC) of ceftazidime/avibactam after 24h were selected for genome sequencing. Three S1 isolates – resistant to 16/4, 32/4 and 64/4 mg/mL of ceftazidime/avibactam, respectively – , two S2 isolates – resistant to 16/4 and 32/4 mg/mL, respectively – , two S3 isolates – resistant to 16/4 and 64/4 mg/mL , respectively – and one S4 isolate – resistant to 16/4 mg/mL of ceftazidime/avibactam – were sequenced. SNPs analysis of these isolates versus the hybrid assembly (S1, S4) or *de novo* Illumina assembly (S2, S3) of the original strains – whose MIC was determined at 16/4 mg/L of ceftazidime/avibactam – was performed. No SNPs were found coding for β-lactamases, porins, efflux pumps nor PBPs related genes (**Supplementary Table 5**).

**REFERENCES**

1. Andrews S. 2010. FastQC: A Quality Control Tool for High Throughput Sequence Data. http://www.bioinformatics.babraham.ac.uk/projects/fastqc/.

2. Bolger AM, Lohse M, Usadel B. 2014. Trimmomatic: A flexible trimmer for Illumina sequence data. Bioinformatics 30.

3. Prjibelski A, Antipov D, Meleshko D, Lapidus A, Korobeynikov A. 2020. Using SPAdes De Novo Assembler. Curr Protoc Bioinforma 70.

4. Brettin T, Davis JJ, Disz T, Edwards RA, Gerdes S, Olsen GJ, Olson R, Overbeek R, Parrello B, Pusch GD, Shukla M, Thomason JA, Stevens R, Vonstein V, Wattam AR, Xia F. 2015. RASTtk: A modular and extensible implementation of the RAST algorithm for building custom annotation pipelines and annotating batches of genomes. Sci Rep 5.

5. Alock BP, Huynh W, Chalil R, Smith KW, Raphenya AR, Wlodarski MA, Edalatmand A, Petkau A, Syed SAS, Tsang KK, Baker SJC, Dave M, McCarthy MC, Mukiri KM, Nasir JA, Golbon B, Imtiaz H, Jiang X, Kaur K, Kwong M, Liang ZCL, Niu KC, Shan P, Yang JYJ, Gray KL, Hoad GR, Jia B, Bhando T, Carfrae LA, Farha MA, French S, Gordzevich R, Rachwalski K, Tu MM, Bordeleau E, Dooley D, Griffiths E, Zubyk HL, Brown ED, Maguire F, Beiko RG, Hsiao WWL, Brinkman FSL, Domselaar G Van, McArthur AG. 2023. CARD 2023: Expanded Curation, Support for Machine Learning, and Resistome Prediction at the Comprehensive Antibiotic Resistance Database. Nucleic Acids Res.

6. Chen L, Yang J, Yu J, Yao Z, Sun L, Shen Y, Jin Q. 2005. VFDB: a reference database for bacterial virulence factors. Nucleic Acids Res Jan:33.

7. Wick R. 2017. Porechop. GitHub Repos. https://github.com/rrwick/Porechop.

8. Wick RR, Judd LM, Gorrie CL, Holt KE. 2017. Unicycler: resolving bacterial genome assemblies from short and long sequencing reads. PLOS Comput Biol 13:1–22.

9. Sullivan MJ, Petty NK, Beatson SA. 2011. Easyfig: A genome comparison visualizer. Bioinformatics 27.

10. Blanco-Martín T, Alonso-García I, González-Pinto L, Outeda-García M, Guijarro-Sánchez P, López-Hernández I, Pérez-Vázquez M, Aracil B, López-Cerero L, Fraile-Ribot P, Oliver A, Vázquez-Ucha JC, Beceiro A, Bou G, Arca-Suárez J, GEMARA/SEIMC-CIBERINFEC Study Group on the activity and resistance mechanisms to new β-lactams and β-lactamase inhibitors (PROTECT). 2024. Activity of cefiderocol and innovative β-lactam/β-lactamase inhibitor combinations against isogenic strains of Escherichia coli expressing single and double β-lactamases under high and low permeability conditions. Int J Antimicrob Agents 63:107150.

11. CLSI. 2020. Performance Standards for Antimicrobial Susceptibility Testing, 30th ed. CLSI supplement M100. Wayne PA: Clinical and Laboratory Standards Institute.

12. Ortiz-Padilla M, Portillo-Calderón I, De Gregorio-Iaria B, Blázquez J, Rodríguez-Baño J, Pascual A, Rodríguez-Martínez JM, Docobo-Pérez F. 2021. Interplay among Different Fosfomycin Resistance Mechanisms in Klebsiella pneumoniae https://doi.org/10.1128/AAC.

**Supplementary Table 1.** Virulence genes displayed by each isolate.

| Isolate | Antiphagocytosis | Adhesion | | Siderophore | | | | Efflux  pumps | Allantoin | Regulation | Toxins | Secretion systems | | | Serum resistance | Hypermucoidy |
| --- | --- | --- | --- | --- | --- | --- | --- | --- | --- | --- | --- | --- | --- | --- | --- | --- |
|  | **Capsule** | **Type 3 fimbriae** | **Type I fimbriae** | **Aerobactin** | **Enterobactin** | **Salmochelin** | **Yersiniabactin** |  |  |  |  | **T6SS-I** | **T6SS-II** | **T6SS-III** | **LPS rfb locus** | **rmpADC/**  **rpmA2** |
| S1 | 14 orfs | *mrkA; B; C; D; F; H; I; J* | *fimA; B; C; D; E; F; G; H; I; K* | *iutA* | *entA; B; C; D; E; F; S; fepA; B; C; D; G; fes* | *iroE; N* | - | *acrA; acrB* | - | *rcsA; rcsB* | - | 3 orfs + *clpV/tssH* (2 orfs); *dotU/tssL; hcp/tssD; icmF/tssM; impA/tssA; ompA; sciN/tssJ; tli1* (5 orfs); *tssF; tssG; vasE/tssK; vgrG/tssI* (2 orfs); *vipA/tssB; vipB/tssC* | *clpV* | 1 orf + *dotU; icmF; impA; F; G; H; J; ompA; sciN* | 7 orfs | - |
| S2 | 14 orfs | *mrkA; B; C; D; F; H; I; J* | *fimA; B; C; D; E; F; G; H; I; K* | *iutA* | *entA; B; C; D; E; F; S; fepA; B; C; D; G; fes* | *iroE; N* | - | *acrA; acrB* | - | *rcsA; rcsB* | - | 3 orfs + *clpV/tssH* (2 orfs); *dotU/tssL; hcp/tssD; icmF/tssM; impA/tssA; ompA; sciN/tssJ; tli1* (5 orfs); *tssF; tssG; vasE/tssK; vgrG/tssI* (2 orfs); *vipA/tssB; vipB/tssC* | *clpV* | 1 orf + *dotU; icmF; impA; F; G; H; J; ompA; sciN* | 7 orfs | - |
| S3 | 14 orfs | *mrkA; B; C; D; F; H; I; J* | *fimA; B; C; D; E; F; G; H; I; K* | *iutA* | *entA; B; C; D; E; F; S; fepA; B; C; D; G; fes* | *iroE; N* | - | *acrA; acrB* | - | *rcsA; rcsB* | - | 3 orfs + *clpV/tssH* (2 orfs); *dotU/tssL; hcp/tssD; icmF/tssM; impA/tssA; ompA; sciN/tssJ; tli1* (5 orfs); *tssF; tssG; vasE/tssK; vgrG/tssI* (2 orfs); *vipA/tssB; vipB/tssC* | *clpV* | 1 orf + *dotU; icmF; impA; F; G; H; J; ompA; sciN* | 7 orfs | - |
| S4 | 14 orfs | *mrkA; B; C; D; F; H; I; J* | *fimA; B; C; D; E; F; G; H; I; K* | *iutA* | *entA; B; C; D; E; F; S; fepA; B; C; D; G; fes* | *iroE; N* | - | *acrA; acrB* | - | *rcsA; rcsB* | - | 3 orfs + *clpV/tssH* (2 orfs); *dotU/tssL; hcp/tssD; icmF/tssM; impA/tssA; ompA; sciN/tssJ; tli1* (5 orfs); *tssF; tssG; vasE/tssK; vgrG/tssI* (2 orfs); *vipA/tssB; vipB/tssC* | *clpV* | 1 orf + *dotU; icmF; impA; F; G; H; J; ompA; sciN* | 7 orfs | - |

**Supplementary Table 2. A)** SNPs matrix among the isolates included in this study. **B)** SNPs per month rate comparison among the isolates from consecutive sampling.

**B)**

| **SNPs comparison** | | | | |
| --- | --- | --- | --- | --- |
| **Isolates**  **A)** | **S1** | **S2** | **S3** | **S4** |
| **S1** | 0 | 9 | 9 | 12 |
| **S2** | 9 | 0 | 11 | 10 |
| **S3** | 9 | 11 | 0 | 19 |
| **S4** | 12 | 10 | 19 | 0 |

| Isolates ^a^ | Nº of SNPs | Months | SNPs/month |
| --- | --- | --- | --- |
| S1 and S3 | 9 | 5 | 1,8 |
| S2 and S3 | 11 | 5 | 2,2 |
| S3 and S4 | 19 | 10 | 1,9 |
| SNPs/month mean |  |  | 2,0 |
| ^a^ S1 and S2 were isolated from the same sample. | | | |

| **Supplementary Table 3.** Population Analysis Profiling (PAP) frequencies of resistant isolates at the selected five concentrations of ceftazidime/avibactam | | | | | | | |
| --- | --- | --- | --- | --- | --- | --- | --- |
|  |  | **Strain** | | | | |  |
|  |  | **S1** | **S2** | **S3** | **S4** |  |  |
| **CZA concentration** | **4/4** | 8,48E-01 | 9,83E-06 | 2,63E-01 | 1,23E-02 |  |  |
|  | **8/4** | 3,38E-04 | - | 2,01E-04 | 2,22E-05 |  |  |
|  | **16/4** | 2,21E-05 | - | 4,60E-06 | 7,78E-07 |  |  |
|  | **32/4** | 9,66E-07 | - | - | - |  |  |
|  | **64/4** | - | - | - | - |  |  |
| CZA, ceftazidime/avibactam | | | | | | | |

| **Supplementary Table 4.** Time-kill analysis results for all isolates at the selected five concentrations of ceftazidime/avibactam | | | | | | | | | | | | | | | | | |
| --- | --- | --- | --- | --- | --- | --- | --- | --- | --- | --- | --- | --- | --- | --- | --- | --- | --- |
|  | | **Strain** | | | | | | | | | | | | | | | |
|  | | **S1** | | | | **S2** | | | | **S3** | | | | **S4** | | | |
| **Time (h)** | | 2 | 4 | 6 | 24 | 2 | 4 | 6 | 24 | 2 | 4 | 6 | 24 | 2 | 4 | 6 | 24 |
| **CZA concentration** | **4/4** | -0.54 | -1.29 | -2.11 | -2.44 | -0.39 | -0.02 | 0.36 | -1.59 | 0.09 | -0.39 | -1.98 | -3.02 | -0.27 | -0.45 | -1.47 | -3.04 |
|  | **8/4** | -0.32 | -1.13 | -1.47 | -2.36 | 0.21 | 0.90 | 2.03 | 1.52 | -0.06 | 0.16 | -1.42 | -2.93 | 0.11 | 0.74 | 1.55 | -2.40 |
|  | **16/4** | -0.35 | 0.59 | 1.76 | -1.90 | 0.31 | 1.32 | 2.42 | 3.66 | -0.46 | 0.87 | 1.71 | -2.53 | -0.20 | 1.63 | 2.05 | -1.60 |
|  | **32/4** | -0.54 | 1.80 | 2.72 | 3.20 | -0.23 | 1.45 | 1.93 | 4.00 | -0.67 | 1.45 | 2.21 | 3.62 | -0.12 | 2.08 | 2.09 | 3.77 |
|  | **64/4** | -0.03 | 2.23 | 2.84 | 2.27 | -0.08 | 1.77 | 2.44 | 3.98 | -0.65 | 2.03 | 2.36 | 3.53 | 0.08 | 2.34 | 2.71 | 3.77 |
| CZA, ceftazidime/avibactam  Values represent the difference (log10 CFU/mL) of each bacterial concentration regarding the initial time point (0h). Red represents a decrease of the bacterial population. Green represents a regrow. | | | | | | | | | | | | | | | | | |
